# USP52 inhibits cell ferroptosis *via* Hippo–YAP pathway and blocks immunotherapy in colorectal cancer

**DOI:** 10.1016/j.jbc.2025.110725

**Published:** 2025-09-15

**Authors:** Jingkai Zhou, Haihang Nie, Chenhui Liu, Lu Ding, Ting Zheng, Li Du, Yali Yu, Yuntian Hong, Chao Yang, Qiu Zhao, Meng Zhang, Haizhou Wang, Fan Wang

**Affiliations:** 1 Department of Gastroenterology, Zhongnan Hospital of Wuhan University, Wuhan, China; 2 Hubei Provincial Clinical Research Center for Intestinal and Colorectal Diseases, Wuhan, China; 3 Hubei Key Laboratory of Intestinal and Colorectal Diseases, Zhongnan Hospital of Wuhan University, Wuhan, China; 4 Department of Gastroenterology, Taikang Tongji (Wuhan) Hospital, Wuhan, China; 5 Department of Anesthesiology, Zhongnan Hospital of Wuhan University, Wuhan, China; 6 Office of Academic Research, Zhongnan Hospital of Wuhan University, Wuhan, China; 7 Department of Endocrinology, Zhongnan Hospital of Wuhan University, Wuhan, China; 8 Department of Pharmacy, Wuhan Seventh Hospital, Wuhan, China; 9 Department of Radiology, Xiangyang Central Hospital, Affiliated Hospital of Hubei University of Arts and Science, Xiangyang, China

**Keywords:** ferroptosis, Hippo/YAP pathway, immunotherapy, ubiquitin specific peptidase, colorectal cancer

## Abstract

Colorectal cancer (CRC) is one of the most prevalent malignancies in humans. Understanding its molecular mechanisms to guide clinical management is crucial. Ferroptosis represents a novel form of regulated cell death that is characterized by highly iron-dependent lipid peroxidation. Despite growing interest, the roles and vulnerabilities determining ferroptosis sensitivity in CRC remain unclear. In this study, we identified ubiquitin-specific peptidase 52 (USP52) as a specific deubiquitinating enzyme of Yes-associated protein (YAP) in CRC, which could stabilize YAP by removing the K11-linked ubiquitin chains. USP52 knockdown decreased the expression of YAP protein and its target gene (CTGF, CYR61). Through a series of comprehensive *in vivo* and *in vitro* experiments, we proved that USP52 promoted CRC cell proliferation, migration, and invasion and attenuated the sensitivity of CRC cells to ferroptosis. Notably, USP52 inhibition retarded tumor growth and enhanced CD8+ T-cell infiltration, which potentiated tumor response to anti–programmed death-ligand-1 immunotherapy *in vivo*. In general, our research uncovered that USP52 suppressed ferroptosis through the Hippo–YAP signaling and highlighted targeting USP52 as a potential therapeutic strategy to boost ferroptosis for enhancing cancer immunotherapy.

Colorectal cancer (CRC) is the third most commonly diagnosed malignancy globally and the second leading cause of cancer-related mortality (1). In 2022, deaths from CRC accounted for nearly one-tenth of all cancer-related deaths. Projections indicate that by 2040, the disease burden associated with CRC will surge by 63% (2). Notably, the incidence of CRC is showing a trend toward younger age groups, with the number of patients under 50 years old steadily increasing year by year (3). Although CRC screening has been demonstrated to reduce the risk of CRC-related mortality (4), participation and its associated benefits remain constrained by factors, such as economic costs, patient awareness, and the possibility of complications. Therefore, there is an urgent need for a comprehensive investigation into the molecular mechanisms underpinning CRC development to identify potential biomarkers and therapeutic targets.

Ubiquitination and deubiquitination modify substrate proteins, regulating their lifespan and function, and play extensive roles in various physiological processes, such as cell proliferation, apoptosis, autophagy, endocytosis, DNA damage repair, and immune responses (5). Ubiquitination and deubiquitination are closely associated with cancer development, gradually emerging as a new target for cancer drug development (6). Deubiquitination is catalyzed by deubiquitinating enzymes (DUBs), which remove ubiquitin from ubiquitinated proteins, thus reversing the ubiquitination process (7). The ubiquitin-specific protease (USP) constituted the majority of DUBs (8). USP52, also known as poly(A)-specific ribonuclease subunit 2, is a member of the USP family (9). A recent study has found that USP52 promoted breast carcinogenesis and reduced the susceptibility of tumor cells to DNA damage by stabilizing the histone chaperone ASF1A (10). In addition, USP52 regulated DNA end resection and chemosensitivity through removing inhibitory ubiquitination from CtIP (11). The latest research has revealed that USP52 promoted bladder cancer progression by modulating ferroptosis through stabilizing SLC7A11/xCT (12). Our previous preliminary study found that USP52 was highly expressed in CRC and was associated with poor prognosis (13). However, we were unable to further explore its underlying molecular mechanisms.

Ferroptosis is a unique form of regulated cell death that involves cellular lipid peroxidation and iron metabolism (14, 15, 16). Lipid metabolic pathways that generate lipid peroxides, when combined with excessive iron ions, trigger ferroptosis, a type of cell death. Lipid peroxides accumulate when their removal mechanism, such as the glutathione-dependent antioxidant system (glutathione peroxidase 4 [GPX4] enzyme), is impaired. This holds potential therapeutic significance for cancer treatment (17, 18). Substantial evidence suggested that the induction of ferroptosis may play a crucial role, either alone or in combination, in tumor suppression (19). Understanding the molecular mechanisms of ferroptosis, including iron metabolism, lipid peroxidation, and antioxidant defenses, could facilitate the development of combination therapies with existing cancer treatments (20, 21). Although the dysregulation of ferroptosis in cancer cells is emerging as a promising target for cancer therapy (22), the role and molecular mechanisms of ferroptosis in CRC remain limited.

The Hippo pathway is a critical regulatory network that governs cell proliferation, differentiation, tissue development, and immune homeostasis. At its core, the Yes-associated protein (YAP) plays a pivotal role in this pathway (23, 24). When the Hippo pathway is inactivated, the phosphorylation of MST1/2 and LATS1/2 is suppressed, leading to reduced phosphorylation of YAP. Consequently, YAP translocates to the nucleus, where it interacts with transcriptional enhancer activator domain 1–4 (TEAD1–4) transcription factors to initiate the transcription of downstream genes (25, 26). In recent years, several studies have discovered that YAP, the Hippo pathway effector, plays a crucial role in regulating ferroptosis (27). Some research has reported that YAP can promote the occurrence of ferroptosis. For instance, Gu *et al.* (28) have found that CYLD promoted ferroptosis through Hippo–YAP signaling, thereby inhibiting prostate cancer progression. Meanwhile, it is reported that YAP could inhibit ferroptosis under certain circumstances. SUFU has been shown to suppress ferroptosis sensitivity in breast cancer cells *via* the Hippo–YAP pathway (29). Therefore, the relationship between YAP and ferroptosis largely depends on a specific cellular and molecular context. However, the molecular mechanisms by which YAP regulates ferroptosis in CRC have not been fully elucidated.

The development of immunotherapy has revolutionized cancer treatment over the past decade. Instead of directly killing cancer cells, immune checkpoint inhibitors (ICIs) activate stalled antitumor immune responses by targeting immune cells. ICIs encompass antibodies that block programmed death-1/programmed death-ligand-1 (PD-1/PD-L1), cytotoxic T lymphocyte antigen-4 (CTLA-4), and lymphocyte activation gene-3 (LAG-3) (30). Nowadays, ICIs, particularly those targeting PD-1 and PD-L1, have been approved for treating various cancers (31, 32), achieving remarkable success in some solid tumors, such as lung cancer and melanoma (33, 34). However, only about one-third of patients respond to ICIs, with nearly all others developing resistance. One of the primary factors contributing to primary resistance to ICIs is the changes of tumor microenvironment (TME) (35, 36). Some studies have reported that ferroptosis could transform an immunosuppressive TME into an inflammatory one (37, 38). Therefore, the application of ferroptosis inducers may help reverse primary resistance to immunotherapy and enhance the efficacy of ICIs (39, 40). However, current research in this area is relatively limited.

Our study was the first to demonstrate that USP52 suppressed ferroptosis and reduced immunotherapy efficacy in CRC by interacting with and deubiquitinating YAP. Combined USP52 depletion and ferroptosis inducers as a novel therapeutic target for enhancing immunotherapy efficacy in CRC.

## Results

### USP52 depletion enhanced the sensitivity of CRC cells to ferroptosis

**Figure 1:**
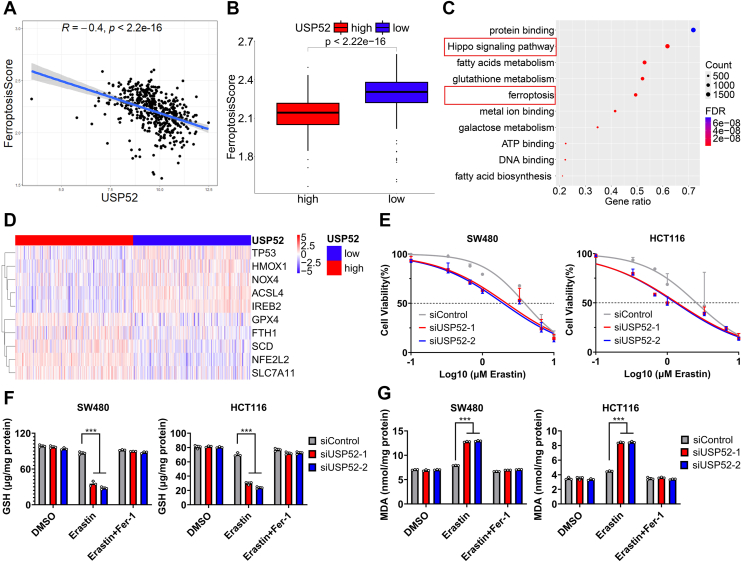
**USP52 inhibited ferroptosis.***A*, correlation analysis revealed a negative correlation between USP52 and the ferroptosis score. *B*, the ferroptosis score decreased in high-USP52 group compared with low-USP52 group. *C*, enrichment analysis indicated that USP52 was closely associated with ferroptosis and the Hippo pathway. *D*, some ferroptosis-related genes exhibited differential expression between USP52-high and USP52-low groups. *E*, cell viability assays demonstrated that USP52-silenced SW480 and HCT116 cells increased the sensitivity to erastin-induced ferroptosis. *F*, GSH assays showed that USP52 knockdown significantly decreased the intracellular GSH level, and this effect can be reversed by Fer-1. *G*, MDA assays revealed that USP52 depletion markedly increased the intracellular MDA level, and this role can be attenuated by Fer-1. ∗∗∗*p* < 0.001. Fer-1, ferrostatin-1; MDA, malondialdehyde; USP52, ubiquitin-specific peptidase 52.

Given that cell death had been reported to play a significant role in tumor therapy in recent years (41). In addition, previous research has explored the regulatory impact of USP52 on ferroptosis in bladder cancer (12). Consequently, we decided to investigate the potential association between USP52 and ferroptosis. As illustrated in Figure 1*A*, USP52 expression was negatively correlated with ferroptosis score. Furthermore, the ferroptosis score was significantly lower in USP52-high groups compared with USP52-low groups (Fig. 1*B*). To elucidate the specific metabolic pathways and genes associated with ferroptosis induced by USP52, we performed enrichment and differential analysis between USP52 subgroups from The Cancer Genome Atlas (TCGA) database. Functional enrichment analysis revealed a significant impact of USP52 on the ferroptosis pathway (Fig. 1*C*). Moreover, numerous ferroptosis-related genes exhibited differential expression between USP52-high and USP52-low groups (Fig. 1*D*). Based on our findings from bioinformatics analysis, we hypothesized that USP52 may exert a potential effect on ferroptosis in CRC. To further explore the functional correlation between USP52 and ferroptosis, we generated transiently USP52-silenced CRC cells with independent siRNAs. The knockdown efficiency was confirmed at the transcriptional and translational levels (Fig. S1, *A* and *B*). Functionally, USP52 knockdown sensitized cancer cells to erastin-induced ferroptosis (Fig. 1*E*). An evident reduction in USP52 significantly enhanced erastin-induced growth inhibition, which was fully reversed by the ferroptosis inhibitor ferrostatin-1 (Fer-1) (Fig. S1*C*). Subsequently, we assessed the levels of GSH, a crucial substrate of GPX4, and malondialdehyde (MDA), a lipid peroxide product. The results showed that the GSH content in SW480 and HCT116 cells significantly decreased after USP52 inhibition, which was fully reversed by Fer-1 (Fig. 1*F*). In addition, the intracellular MDA levels markedly increased upon downregulation of USP52, which was partially attenuated by Fer-1 (Fig. 1*G*). Collectively, the preceding results indicate that USP52 inhibition enhances the sensitivity of CRC cell lines to ferroptosis.

### USP52 promoted cell proliferation, migration, and invasion

**Figure 2:**
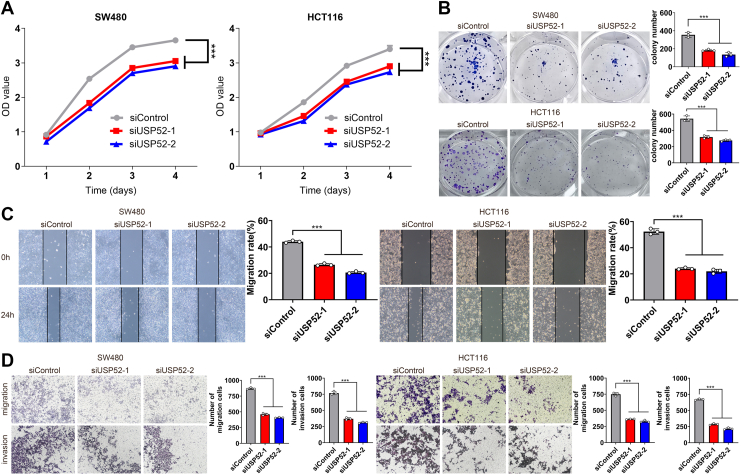
**USP52 promoted the proliferation, migration, and invasion of CRC cells.***A*, the CCK-8 assay demonstrated that USP52 knockdown significantly inhibited the proliferation of SW480 and HCT116 cells. *B*, the colony formation assay revealed that the number of colonies was markedly reduced after USP52 depletion. *C*, the wound healing assay indicated that USP52 knockdown significantly inhibited cell migration. *D*, the transwell assay found that USP52 depletion markedly inhibited cell migration and invasion. ∗∗∗*p* < 0.001. CCK-8, Cell Counting Kit-8; CRC, colorectal cancer; USP52, ubiquitin-specific peptidase 52.

To further elucidate the role of USP52 in CRC phenotypes, we conducted a series of experiments. Initially, the Cell Counting Kit-8 (CCK-8) assay demonstrated that USP52 depletion significantly suppressed the proliferation of SW480 and HCT116 cells (Fig. 2*A*). Similarly, the colony formation assay indicated that USP52 knockdown noticeably decreased the number of SW480 and HCT116 cell colonies (Fig. 2*B*). Furthermore, the wound healing assays and the transwell assays revealed that USP52 inhibition impaired the migration and invasion capacity of SW480 and HCT116 cells (Fig. 2, *C* and *D*). The aforementioned results showed that USP52 is essential for CRC cell survival and progression.

### USP52 regulated the Hippo–YAP axis

**Figure 3:**
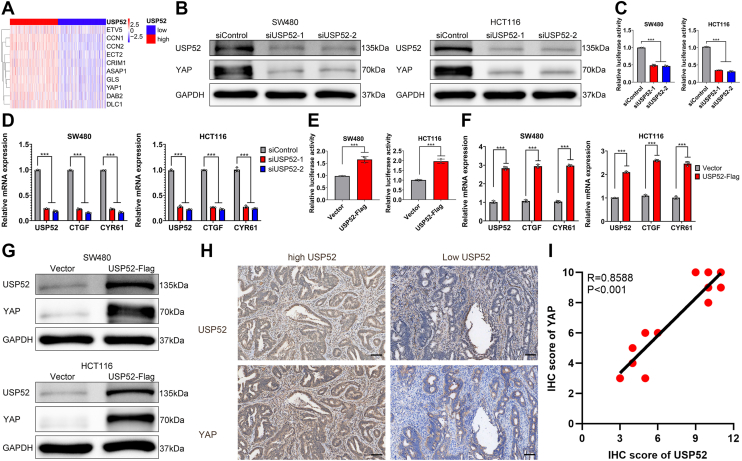
**USP52 regulated the Hippo–YAP pathway.***A*, the expressions of YAP1, CCN1, and CCN2 were upregulated in high-USP52 group. *B*, USP52 knockdown led to a significant decrease in YAP expression at the translational level. *C*, the luciferase reporter assay revealed that USP52 inhibition suppressed TEAD response element activity. *D*, USP52 depletion resulted in an evident reduction in YAP-targeted gene (CTGF, CYR61) expression at the transcriptional level. *E*, the luciferase reporter assay revealed that USP52 overexpression promoted TEAD response element activity. *F*, USP52 overexpression resulted in an obvious increase in YAP-targeted gene (CTGF, CYR61) expression at the transcriptional level. *G*, USP52 overexpression led to an evident increase in YAP expression at the translational level. *H*, IHC analysis indicated a positive correlation between USP52 and YAP (the scale represents 100 μm). *I*, quantitative analysis of IHC confirmed a positive correlation between USP52 and YAP. ∗∗∗*p* < 0.001. IHC, immunohistochemistry; TEAD, transcriptional enhancer activator domain; USP52, ubiquitin-specific peptidase 52; YAP, Yes-associated protein.

The aforementioned enrichment analysis demonstrated a significant enrichment of the Hippo–YAP pathway (Fig. 1*C*). Importantly, previous studies have reported that multiple DUBs are enabled to modulate YAP expression in gastrointestinal cancer (42, 43). Therefore, with keen interest, we examined the expression of USP52, YAP, and its targeted genes from the TCGA database. Our analysis uncovered a positive correlation between USP52 and YAP1 as well as its targeted genes CCN1 (CYR61) and CCN2 (CTGF) in the TCGA-Colon Adenocarcinoma (COAD) cohort (Fig. 3*A*). To further confirm whether USP52 affected YAP expression, we silenced USP52 in CRC cells for subsequent investigation. The result of Western blot (WB) analysis indicated that USP52 depletion decreased the expression of YAP protein at the translational level in SW480 and HCT116 cells (Fig. 3*B*). In addition, the luciferase reporter assay demonstrated that USP52 inhibition suppressed TEAD response element activity in CRC cells (Fig. 3*C*). Furthermore, quantitative PCR (qPCR) analysis showed that USP52 knockdown suppressed the transcription of YAP-targeted genes, including CTGF and CYR61, in these cell lines (Fig. 3*D*). Moreover, data from the TCGA database displayed that USP52 was positively associated with YAP1 and its targeted genes (CTGF and CYR61) (Fig. S2, *A*–*C*). In addition, the nuclear and cytoplasmic fractionation experiments further showed that USP52 knockdown resulted in an evident decrease in YAP expression in the cytoplasm and nucleus (Fig. S2*D*). To further validate our findings, we overexpressed USP52 in SW480 and HCT116 cells. The luciferase reporter assay showed that USP52 overexpression enhanced TEAD response element activity (Fig. 3*E*). Moreover, the qPCR data showed that USP52 overexpression enhanced the expression of Hippo target genes, including CTGF and CYR61 (Fig. 3*F*). The WB analysis also confirmed that USP52 overexpression enhanced YAP protein levels (Fig. 3*G*). Finally, we observed a positive correlation between the expression levels of USP52 and YAP protein (Fig. 3, *H* and *I*). All these data indicated that USP52 positively regulates YAP in CRC cells.

### USP52 facilitated CRC progression *via* Hippo–YAP signaling

**Figure 4:**
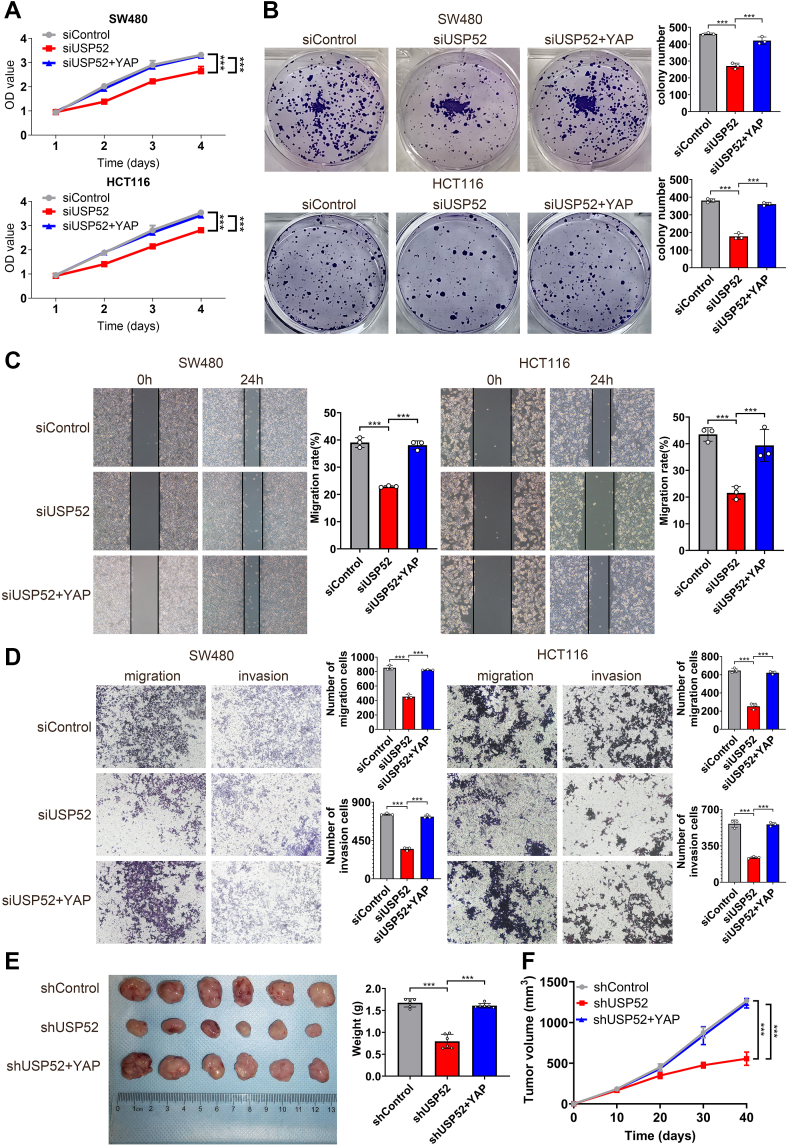
**USP52 promoted CRC progression through the Hippo–YAP signaling.***A*, the CCK-8 assay demonstrated that USP52 knockdown significantly inhibited the proliferation of SW480 and HCT116 cells, and this effect could be reversed by YAP overexpression. *B*, the colony formation assay revealed that USP52 inhibition markedly reduced the number of colonies, which could be attenuated by YAP overexpression. *C*, the wound healing assay indicated that USP52 depletion significantly inhibited cell migration, which could be partially rescued by YAP overexpression. *D*, the transwell assay found that USP52 knockdown noticeably inhibited cell migration and invasion, and this effect could be partially rescued by YAP overexpression. *E*, the xenograft mouse model showed that USP52 depletion significantly decreased tumor size and weight, and this effect was reversed by YAP overexpression. *F*, the tumor volume was markedly reduced after USP52 knockdown, which was partially attenuated by YAP overexpression. ∗∗∗*p* < 0.001. CCK-8, Cell Counting Kit-8; CRC, colorectal cancer; USP52, ubiquitin-specific peptidase 52; YAP, Yes-associated protein.

Since we proved that USP52 was required for CRC progression and Hippo–YAP activation, we carried out further rescue experiments to test whether USP52 modulated cancer progression *via* YAP. The CCK-8 assay indicated that USP52 depletion noticeably suppressed CRC growth, whereas YAP overexpression partially attenuated this growth inhibition effect (Fig. 4*A*). Consistently, the colony formation assay showed that the reduced clone numbers caused by USP52 depletion could be partially rescued by YAP overexpression (Fig. 4*B*). Moreover, in the wound healing and transwell assay, the impaired migration and invasion capacity induced by USP52 depletion could be partially recovered by YAP overexpression in SW480 and HCT116 cells (Fig. 4, *C* and *D*). Furthermore, we have also designed rescue experiments by suppressing YAP in USP52 overexpression cells. The results proved that USP52 overexpression promoted CRC cells proliferation, migration, and invasion, which could be attenuated by YAP depletion (Fig. S3, *A*–*D*). To further evaluate this pathway *in vivo*, we established the xenograft mouse model. The results showed that USP52 depletion inhibited tumor growth, and this effect was reversed by YAP overexpression (Fig. 4, *E* and *F*). The aforementioned results showed that USP52 promotes CRC progression *via* YAP.

### USP52 stabilized the YAP protein by inhibiting the K11-linked polyubiquitination of YAP

**Figure 5:**
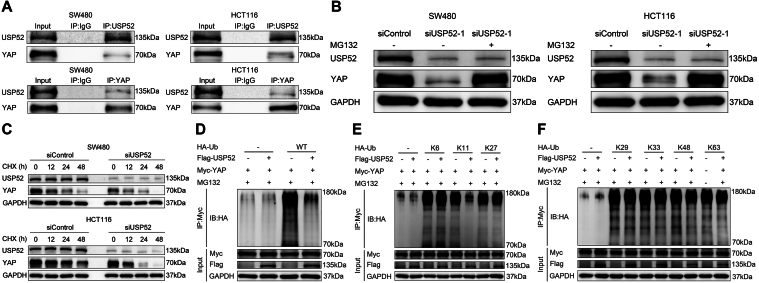
**USP52 interacted with YAP and decreased the K11-linked polyubiquitination of YAP.***A*, endogenous IP experiments demonstrated that USP52 interacted with YAP. *B*, USP52 depletion reduced the YAP protein expression, and this effect could be reversed by MG132. *C*, USP52 knockdown decreased the stability of YAP in SW480 and HCT116 cells. *D*, the ubiquitination assay demonstrated that USP52 overexpression inhibited YAP total polyubiquitination in HEK293 cells. *E* and *F*, in ubiquitination assay, USP52 overexpression significantly suppressed K11-linked ubiquitination of YAP and, to a less degree, K48- and K63-linked ubiquitination in HEK293 cells. HEK293, human embryonic kidney 293 cell line; IP, immunoprecipitation; USP52, ubiquitin-specific peptidase 52; YAP, Yes-associated protein.

Since USP52 modulates CRC progression through the Hippo–YAP axis, we further investigated the underlying regulatory mechanism. The endogenous immunoprecipitation assay indicated that USP52 could combine with YAP in CRC cells (Fig. 5*A*). Since YAP degradation relies on the effect of the proteasome, we utilized the proteasome inhibitor MG132. USP52 depletion decreased YAP protein levels, whereas MG132 treatment diminished this decrease in YAP protein levels (Fig. 5*B*). The protein half-life assay demonstrated that USP52 reduction impaired YAP protein stability in CRC cells (Fig. 5*C*). Since USP52 belongs to the deubiquitinase family, we subsequently assessed the role of USP52 in YAP ubiquitination. The ubiquitination assay indicated that USP52 overexpression could significantly decrease the total YAP ubiquitination level (Fig. 5*D*). Eight different types of polyubiquitination linkages have been reported, seven of which are linked to seven conserved lysine residues, namely, K6 (Lys6), K11 (Lys11), K27 (Lys27), K29 (Lys29), K33 (Lys33), K48 (Lys48), and K63 (Lys63) (44). Subsequently, we further explored which subtypes of ubiquitin chains were involved in YAP modification and were regulated by USP52. Our research showed that USP52 overexpression markedly inhibited K11-linked ubiquitination of YAP, and, to a less extent, K48- and K63-linked ubiquitination. Conversely, USP52 overexpression had no appreciable effect on the ubiquitination of YAP with K6, K27, K29, and K33 linkages (Fig. 5, *E* and *F*).

### USP52 regulated ferroptosis by regulating the Hipp–YAP pathway

**Figure 6:**
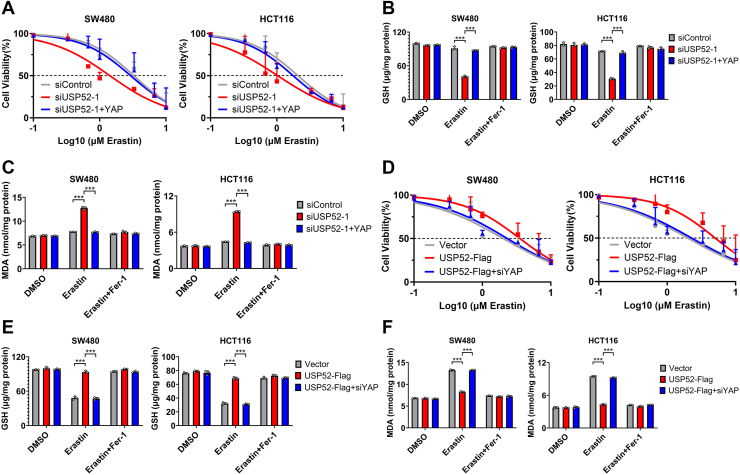
**USP52 inhibited ferroptosis through the Hippo–YAP axis *in vitro*.***A*, cell viability assays revealed that USP52-slienced SW480 and HCT116 cells increased the sensitivity to erastin-induced ferroptosis, and this effect could be reversed by YAP overexpression. *B*, GSH assays showed that USP52 knockdown significantly decreased intracellular GSH levels, which could be partially rescued by YAP overexpression. *C*, MDA assays demonstrated that USP52 inhibition markedly increased intracellular MDA levels, which was partially attenuated by YAP overexpression. *D*, cell viability assays revealed that USP52 overexpression decreased the sensitivity of SW480 and HCT116 cells to erastin-induced ferroptosis, and this effect could be reversed by YAP knockdown. *E*, GSH assays indicated that USP52 overexpression noticeably increased intracellular GSH levels, and this effect could be partially rescued by YAP inhibition. *F*, MDA assays showed that USP52 overexpression significantly decreased intracellular MDA levels, which could be partially reversed by YAP depletion. ∗∗∗*p* < 0.001. MDA, malondialdehyde; USP52, ubiquitin-specific peptidase 52; YAP, Yes-associated protein.

Given that multiple studies have demonstrated that YAP enables tdirect regulation of ferroptosis in epithelial cells (28, 29), we further investigated whether USP52 regulated ferroptosis through Hippo–YAP signals. We replenished YAP in USP52 knockdown SW480 and HCT116 cells to assess cell viability and measure intracellular GSH and MDA levels. Whereas USP52 knockdown increased erastin-induced cell ferroptosis and MDA levels, and decreased GSH levels in CRC cells, YAP overexpression rescued these changes (Fig. 6, *A*–*C*). Consistently, USP52 overexpression significantly decreased cell death and MDA levels, and increased GSH levels, which could be partially reversed by further YAP knockdown in CRC cells (Fig. 6, *D*–*F*). To further confirm USP52 could prevent ferroptosis by regulating the Hippo–YAP pathway *in vivo*, we detect the content of MDA and GSH in subcutaneous tumor tissues. The results indicated that USP52 depletion significantly reduced GSH content and increased MDA levels, which was partially attenuated by YAP overexpression in tissues (Fig. S4, *A* and *B*). These findings suggest that USP52 depletion promotes ferroptosis by inhibiting the Hippo–YAP pathway.

### USP52 inhibition sensitized tumors to anti-PD-L1 immunotherapy through ferroptosis

**Figure 7:**
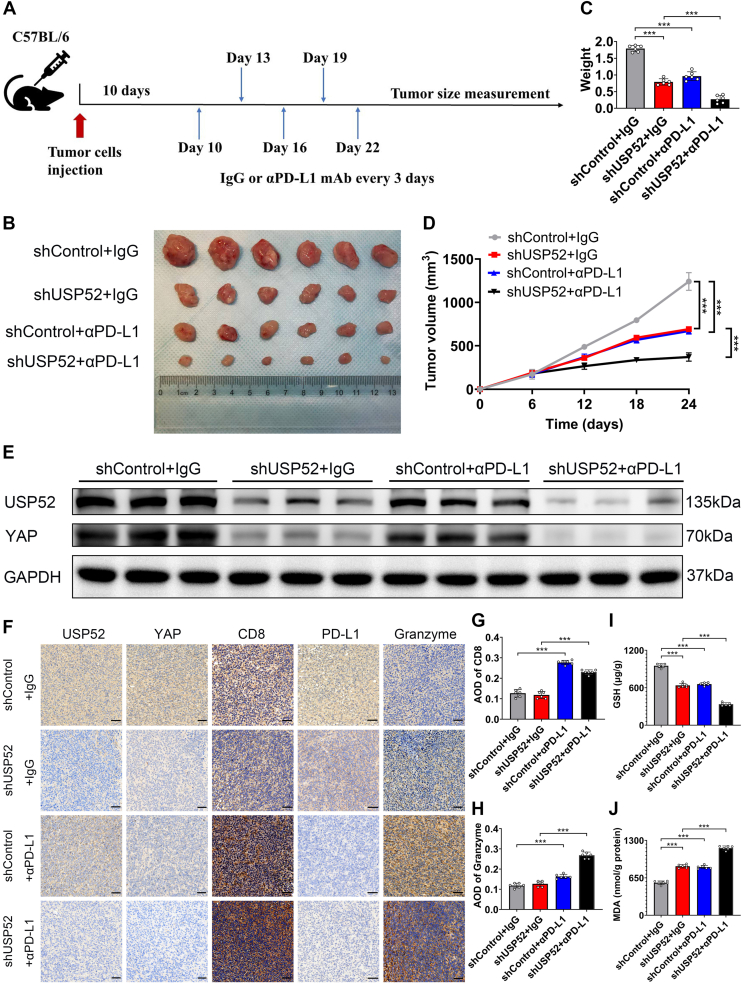
**USP52 attenuated immunotherapy efficacy by inhibiting ferroptosis.***A*, C57 mice were subcutaneously injected with MC38 cells to induce tumor formation. After 10 days, IgG or αPD-L1 treatment was initiated and administered once every 3 days until tissue collection. *B*, in subcutaneous xenograft models, a significant reduction in tumor size was observed following αPD-L1 treatment. *C*, the tumor weight was significantly decreased after anti-PD-L1 treatment. *D*, the tumor volume was markedly reduced after αPD-L1 treatment. *E*, immunoblotting analysis confirmed that the expression levels of both USP52 and YAP were decreased in USP52-silenced tumors. *F*, IHC analysis revealed a significant decrease in the expression of YAP and PD-L1, along with an evident increase in the expression of CD8 and granzyme B, following αPD-L1 treatment (the scale represents 50 μm). *G*, the relative absorbance of CD8 staining was quantified on the basis of AOD. *H*, the quantification of granzyme staining's relative absorbance was carried out using AOD. *I*, GSH assays demonstrated that GSH levels were significantly decreased after αPD-L1 treatment. *J*, MDA assays indicated that MDA levels were significantly increased after αPD-L1 treatment. ∗∗∗*p* < 0.001. AOD, average optical density; CRC, colorectal cancer; IgG, immunoglobulin G; IHC, immunohistochemistry; MDA, malondialdehyde; PD-L1, programmed death-ligand-1; USP52, ubiquitin-specific peptidase 52.

The aforementioned results demonstrated that USP52 inhibition promoted ferroptosis *via* downregulating YAP expression. To investigate whether USP52 inhibition could enhance the efficacy of anti-PD-L1 immunotherapy, we established the murine subcutaneous tumor model. After 10 days, the mice were divided into different groups and subjected to different treatments, as shown in Figure 7*A*. Our results showed that tumor growth was significantly inhibited following treatment, and this effect was further enhanced by USP52 depletion (Fig. 7, *B*–*D*). In addition, immunoblotting analysis verified that both USP52 and YAP exhibited reduced expression levels in USP52-silenced tumors (Fig. 7*E*). To further explore the differences in the immune milieu following the synergistic intervention of USP52 inhibition and αPD-L1, immunohistochemistry (IHC) focusing on CD8+ T cells within tumors was performed. Notably, the CD8+ T-cell infiltration and granzyme levels were significantly increased in tumors derived from the combined treatment modality (Fig. 7, *F*–*H*). Previous studies have reported that ferroptosis can transform an immunosuppressive TME into an inflammatory TME rich in antitumor immune cells, thus synergizing the antitumor immune response (37, 38). Therefore, with great interest, we analyzed the levels of GSH and MDA in different groups. The results revealed that the treatment significantly decreased GSH levels and increased MDA levels, and this effect was further enhanced by USP52 knockdown (Fig. 7, *I* and *J*). These findings indicate that USP52 depletion enhances immunotherapy efficacy by promoting ferroptosis.

## Discussion

**Figure 8:**
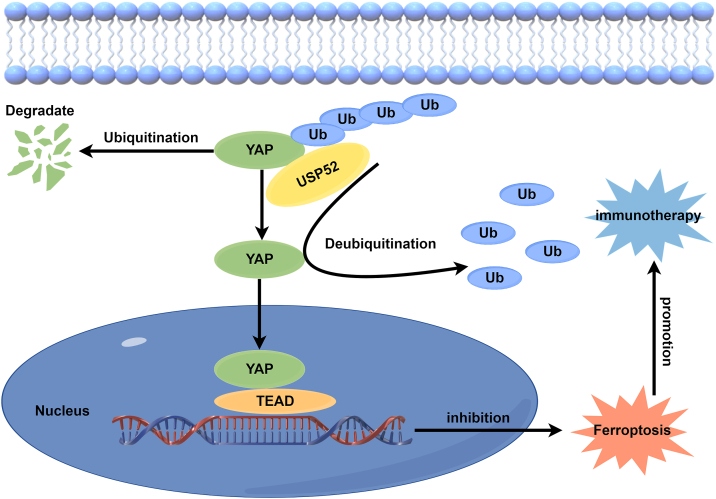
**USP52 interacted with YAP and inhibited YAP K11-linked ubiquitination and degradation in CRC cells, which inhibited ferroptosis and reduced immunotherapy efficacy.** This image was created by Figdraw. CRC, colorectal cancer; USP52, ubiquitin-specific peptidase 52; YAP, Yes-associated protein.

In this study, we discovered that USP52 modulated ferroptosis through the Hippo–YAP axis, thereby influencing immunotherapy. Our findings revealed that USP52 inhibited ferroptosis and exhibited a positive correlation with YAP. USP52 interacts with YAP and inhibits YAP polyubiquitination and breakdown, which further decreases the sensitivity of CRC cells to ferroptosis and ultimately attenuates immunotherapy efficacy (Fig. 8). Based on these findings, we hypothesize that USP52 serves as a critical regulator of ferroptosis and Hippo signaling. Moreover, the combination of USP52 inhibition with ferroptosis inducers may represent a promising therapeutic strategy to enhance immunotherapy for CRC patients.

USP52, also known as poly(A)-specific ribonuclease subunit 2, as a member of the USP family, is capable of inhibiting the ubiquitin-mediated process of protein degradation, thereby maintaining protein stability (9). This process, named deubiquitination, is mediated by DUBs, and most of them belong to the USP family (7, 8). In recent years, the USPs have gradually become a research focus, and USP52 is no exception. Studies have reported that USP52, as a key component of P-bodies, could prevent the degradation of HIF1A (9). Moreover, the involvement of USP52 in tumorigenesis has also been elucidated by relevant studies. In breast cancer, USP52 has been shown to decrease the ubiquitination level of the histone chaperone ASF1A, thereby stabilizing its protein and promoting cell proliferation as well as enhancing DNA damage tolerance (10). In addition, USP52 has been found to decrease cell sensitivity to poly(ADP-ribose) polymerase inhibitor in a CtIP-dependent manner, subsequently promoting cell proliferation in osteosarcoma (11). The latest research has found that USP52 reduced the K48-linked ubiquitination level of xCT and maintained its protein stability, thus promoting bladder cancer progression (12). Similarly, in this study, we discovered that USP52 significantly diminished the K11-linked polyubiquitination level of YAP and stabilized its protein, thereby promoting CRC progression.

Ferroptosis is an iron-dependent form of programmed cell death distinct from apoptosis, necroptosis, and autophagy, characterized by iron dependency and lipid peroxidation. The characteristics of ferroptosis are shown in a significant increase in lipid peroxide levels, such as MDA and 4-hydroxynonenal, a substantial generation of reactive oxygen species, and a marked decrease in GSH levels (45). As a novel form of cell death, ferroptosis offers new strategies for exploring cancer therapies, which has attracted widespread attention and research from researchers (46, 47). Ubiquitination, as one of the important ways for post-translational modification of proteins, plays a crucial role in intracellular protein localization, metabolism, functional regulation, and degradation. It is implicated in regulating nearly all aspects of human cell biology and physiology, including cell cycle, proliferation, apoptosis, differentiation, gene expression, and signal transduction (5). In recent years, numerous studies have established a biological link between ubiquitination and ferroptosis. For instance, USP7 has been shown to suppress ferroptosis by activating stearoyl-CoA desaturase in gastric cancer (48), whereas USP8 knockdown enhanced ferroptosis through inhibiting GPX4 in CRC (49). Consistently, in our study, we observed that USP52 depletion increased the sensitivity of CRC cells to erastin-induced ferroptosis, accompanied by a decrease in intracellular GSH levels and an increase in MDA levels. These findings suggest that USP52 knockdown may promote ferroptosis. However, we failed to further explore the ferroptosis-related molecules regulated by USP52. Recent research has reported that USP52 inhibition promoted ferroptosis by inhibiting SLC7A11/xCT in bladder cancer (12). Therefore, we hypothesize that USP52 may also influence ferroptosis in CRC by regulating xCT. This hypothesis warrants further investigations to determine whether this mechanism is reasonable in CRC cells.

Previous studies have reported that polyubiquitination is mainly associated with proteasome-dependent degradation, activity, and translocation of the substrates (50). As for polyubiquitination, ubiquitin molecules can be linked to each other through seven different receptor sites, since ubiquitin molecules contain seven sites of lysine (K6, K11, K27, K29, K33, K48, and K63) (51). K48-linked polyubiquitin chains are implicated in the proteolysis of certain substrates through the ubiquitin–proteasome system (52). A previous study has reported that USP36 overexpression markedly decreased the K48-linked ubiquitination level of YAP (53). Mo *et al.* (54) have demonstrated that USP40 stabilized YAP by removing the K48-linked polyubiquitin chains. While K63-linked polyubiquitin chains modulate protein activity, localization, and its interaction with other proteins, non-K63 polyubiquitin linkages, particularly K48-linked ubiquitin chains, target proteins for proteasomal degradation (55). One study has identified YAP as a substrate of SKP2 and uncovered SKP2’s role in mediating YAP’s nonproteolytic, K63-linked ubiquitination through the SCF (Skp1–Cul1–F-box protein) complex and UBCH5c (56). Panda *et al.* (57) have reported YAP’s nuclear relocation and transcriptional activity *via* K63-linked YAP polyubiquitination. In addition, another study has revealed that YAP undergoes TRAF6-dependent K63 chain ubiquitination in response to interleukin-1β, which resulted in increased YAP nuclear localization and protein stability (58). K11-linked polyubiquitin chains are recognized by proteasomal receptors for substrate degradation, but the same receptors can also interact with K48-linked chains (59). Research has shown that the K48/K11 branched chains formed by the combination of K11 and K48 can enhance protein degradation by the proteasome (60). Tian *et al.* (43) found that USP19 overexpression largely decreased the K48- and K11-linked polyubiquitination level of YAP by removing the K48- and K11-linked ubiquitin chains from YAP. In addition, ubiquitination modification of other molecules can also be mediated by K11-linked polyubiquitination. One study has indicated that USP1 regulated TAZ protein stability by reducing the K11-linked polyubiquitination of TAZ (61). Wang *et al.* (62) also reported that TRIM3 specifically catalyzed K11-linked polyubiquitination of xCT. Consistent with previous studies, our research has found that USP52 overexpression markedly inhibited K11-linked ubiquitination of YAP.

Accumulating research has elucidated the close association between dysregulation of the Hippo signaling pathway and YAP activation with various types of cancer, including colorectal, liver, and stomach. Dysregulation of the Hippo pathway can result in YAP dephosphorylation and translocation into the nucleus, thereby promoting tumor development (63). In addition, ubiquitination is one of the crucial post-translational modifications on YAP. E3 ligases assist or directly catalyze the transfer of ubiquitin from E2 to the YAP protein and promote its ubiquitination and degradation (64). Conversely, USPs significantly decreased the total polyubiquitination of YAP and stabilized it by removing the ubiquitin chain from YAP, thereby promoting tumor progression (61). For example, USP19 interacted with the WW domain of YAP and reduced the K11- and K48-linked polyubiquitination level of YAP, thus promoting hepatocellular carcinoma progression (43). Similarly, USP36 has been demonstrated to interact with the WW domain of YAP and decrease the K48-linked polyubiquitination level of YAP, thereby promoting esophageal squamous carcinoma progression (53). Moreover, some studies have reported that USP40 is mainly associated with the transactivation domain of YAP and cleaves the K48-linked polyubiquitination of YAP, thus accelerating hepatocellular carcinoma progression (54). In our study, USP52 stabilized YAP by reducing the K11-linked polyubiquitination of YAP, thereby promoting CRC progression. However, we were unable to further explore the specific domain of YAP that interacted with USP52. The YAP protein is composed of the TEAD-binding domain, WW domain, and transactivation domain (65), and USP52 also belongs to the USP family. Therefore, we reasonably hypothesize that USP52 may bind to one of these domains of YAP. It is worth noting that we will conduct further research on the specific domains of interaction between USP52 and YAP.

Previous studies have demonstrated that YAP–TAZ modulate ferroptosis through multiple ways, including regulating polyunsaturated fatty acid with phospholipid synthesis (ACSL4), intracellular iron availability (transferrin receptor), and reactive oxygen species production (NOX2/NOX4) (66). It has been reported that YAP can inhibit ferroptosis. Specifically, YAP–TAZ forms complexes with ATF4, thereby activating SLC7A11 and preventing ferroptosis (67). In addition, activation of the YAP protein has been shown to upregulate the expression of SLC7A11, thus inhibiting ferroptosis in non–small cell lung cancer (68). Consistently, our research also revealed that YAP activation also suppressed ferroptosis. However, we failed to further investigate the ferroptosis-related molecules regulated by YAP. Based on previous research results and our findings, we speculate that USP52 regulates the expression of SLC7A11 *via* the Hippo–YAP axis, thereby inhibiting ferroptosis in CRC.

ICIs have emerged as a primary therapeutic approach for various cancers, including melanoma, lung cancer, and numerous other solid tumors, showcasing significant clinical efficacy (69). The uniqueness of immunotherapy lies in its ability to guide the patient's immune system to generate enduring and persistent responses, ultimately leading to prolonged disease remission (70). However, ICIs also have several drawbacks, including a relatively low clinical response rate and tumor resistance. Studies have indicated that the resistance of tumors to ICIs primarily originates from alterations in the TME (35, 36). Moreover, an immunosuppressive TME promotes angiogenesis and inhibits the antitumor activity of CD8+ T cells, thereby allowing tumor cells to evade detection by the host immune surveillance system (71). Therefore, modifying the composition of TME represents a key pathway for enhancing ICI efficacy. Recent studies have surprisingly revealed that CD8+ T cells can suppress tumors by inducing ferroptosis and pyroptosis (72). It has been reported that ferroptosis can limit the function of immunosuppressive cells, such as tumor-associated macrophages and Treg cells in cold tumors, transforming the immunosuppressive TME into an inflammatory TME rich in antitumor immune cells, including CD8+ T cells (37, 38). Therefore, ferroptosis inducers, including lapatinib, statins, trigonelline, and others, may help reverse primary resistance to immunotherapy and enhance the efficacy of ICIs (39, 40). Currently, studies have reported that USPs affect immunotherapy efficacy by regulating ferroptosis. The inhibition of the USP8–GPX4 axis promoted ferroptosis and enhanced CD8+ T-cell infiltration, thereby improving the efficacy of anti-PD-1 immunotherapy in CRC (49). Consistent with this, our research found that USP52 depletion promoted ferroptosis and increased CD8+ T-cell infiltration, thus enhancing the efficacy of anti-PD-L1 treatment in CRC. The aforementioned research also discovered that the USP8 inhibitor (DUB-IN-2) in combination with the ferroptosis inducer, SAS, significantly retarded the tumor growth and promoted CD8+ T-cell infiltration in the TME, thereby enhancing the effectiveness of anti-PD-1 immunotherapy in CRC (49). Given that USP52 is also a member of the USP family, we hypothesize that USP52 inhibition in combination with the ferroptosis inducer, erastin, can similarly promote CD8+ T-cell infiltration in the TME, thereby increasing the sensitivity of CRC to anti-PD-L1 treatment. Therefore, the further exploration of USP52 inhibitors and their combination with erastin represents a promising strategy to improve immunotherapy efficacy.

In conclusion, our study not only advances our understanding of the role of USP52 in regulating ferroptosis and the Hippo–YAP signaling pathway in CRC but also underscores a potential combined ferroptosis inducer for enhancing immunotherapy.

## Experimental procedures

### TCGA data download

The expression data of CRC patients were downloaded from TCGA Program (https://www.cancer.gov/ccg/research/genome-sequencing/tcga). To analyze the expression of USP52, ferroptosis-related genes, and Hippo–YAP signaling–targeted genes, the gene expression matrix of CRC patients was acquired from TCGA-COAD cohort using appropriate R packages. A correlation plot was generated using the Pearson’s analysis. The heatmap plot was generated using the “pheatmap” R package.

### Correlation analysis between ferroptosis score and USP52 expression

According to the previous study, 60 ferroptosis-related molecules, including ACSL4, GPX4, SLC7A11, transferrin receptor, were selected in the subsequent analyses (73). In TCGA-COAD cohort, we calculated the ferroptosis score using “ssGSEA” R package. In addition, we investigated the correlation between USP52 expression and the ferroptosis score through Pearson's correlation analysis. Both the ferroptosis-related genes and the ferroptosis score can be found in Tables S1 and S2.

### Enrichment analysis

In TCGA-COAD cohort, we divided the patients into high and low USP52 groups based on the median USP52 expression. Then we identified 2569 differentially expressed genes (DEGs) *via* “limma” R package. The criterion for selecting DEGs was set as adjusted *p* < 0.05 and |log (fold change)| value >1.3. Subsequently, we conducted functional enrichment analysis from The Database for Annotation, Visualization, and Integrated Discovery online website (https://davidbioinformatics.nih.gov/). Based on the criterion including false discovery rate <0.05 and count value >280, we selected the top 10 biological functions and signaling pathways. Both the DEGs and enrichment scores can be found in Tables S3 and S4.

### Cell culture and transfection

The CRC cell lines (SW480, HCT116, and MC38) and the human embryonic kidney 293 (HEK293) cells were obtained from the China Center for Type Culture Collection (Wuhan). SW480, MC38, and HEK293 cells were cultured in Dulbecco’s modified Eagle’s medium (Procell) supplemented with 10% fetal bovine serum (FBS; Cell-Box), whereas HCT116 cells were cultured in McCoy's 5A medium (Procell) with 10% FBS in an incubator at 37 °C with 5% CO_2_. The siRNAs targeting USP52 and YAP were purchased from Sangon Biotech. The siRNA sequences were as follows: siUSP52-1: GGAGGACUUUGACUUCAAATT, siUSP52-2: CCAUCAUGAGACAGACAAATT, siYAP-1: GACUGACAGCAGCACUCUATT, and siYAP-2: UUUGAAGUCAAAGUCCUCCTT. In addition, the plasmids were acquired from MiaoLing Biology. The siRNA transfection was performed using Lipofectamine 3000 reagent (Invitrogen), and plasmid transfection was carried out using Lipofectamine 8000 reagent (Beyotime) according to the manufacturer’s instructions.

### RNA extraction, reverse transcription, and quantitative real-time PCR analysis

SW480 and HCT116 cells were seeded in 6-well plates and transfected with siUSP52 or siControl. After 48 h, TRIzol reagent (Invitrogen) was added, and the mixture was lysed on ice for 10 min. Then chloroform was added at a ratio of 5:1 (with 1 ml TRIzol and 200 μl chloroform), and the mixture was lysed on ice for 10 min. The samples were centrifuged at 12,000 rpm for 15 min at 4 °C. The supernatant was collected, mixed with isopropanol at a ratio of 1:1 (with 500 μl supernatant and 500 μl isopropanol), and incubated on ice for 10 min. The samples were centrifugation again at 12,000 rpm for 10 min at 4 °C. The supernatant was discarded, and the pellet was retained. The pellet was washed twice with 75% ethanol, and centrifuged at 12,000 rpm for 5 min at 4 °C. Diethyl pyrocarbonate water was added to dissolve RNA. Reverse transcription was performed using the ReverTra AceTM qPCR RT Kit (TOYOBO), and quantitative real-time PCR was carried out using the UltraSYBR Mixture (Cwbio) according to the manufacturer's instructions. The data were analyzed using the LightCycler system (Roche). The relevant primer sequences are shown in Table S5.

### Protein extraction and concentration determination

The CRC cells were harvested and lysed with NP-40 buffer (Beyotime) containing protease and phosphatase inhibitors (MCE). The samples were lysed on ice for 30 min and then centrifuged at 13,300 rpm for 25 min at 4 °C. The supernatant was collected. Protein content analysis was conducted using the BCA Kit (Beyotime) according to the manufacturer's instructions. The absorbance at 570 nm was measured using a microplate reader.

### Western blot

Equivalent amounts of protein were separated by 10% SDS-PAGE and transferred to polyvinylidene difluoride membranes (Millipore). Then, the membranes were blocked in protein-free quick blocking buffer (X-Blot) for 30 min and incubated with primary antibodies at 4 °C overnight. Afterward, the membranes were washed three times with Tris-buffered saline with Tween and incubated with secondary antibodies for 1 h 30 min. After being washed three times with Tris-buffered saline with Tween, the membranes were probed with an ECL system (Affinity), and images were captured using a Tanon 5200 system (Tanon). GAPDH served as the internal control. The primary antibodies and secondary antibodies used in the present study are shown in Table S6.

### Cell viability assay

SW480 and HCT116 cells were seeded in 6-well plates and transfected with siUSP52 or siControl. After 24 h, the cells were washed and counted. Subsequently, they were seeded in a 96-well plate at a density of 5000 cells per well. After 24 h, we added different concentrations of Erastin (Selleck) and Fer-1 (MCE). CCK-8 (Abbkine) was added after 24 h. After 2 h incubation, the absorbance at 450 nm was measured. The experiments were executed in triplicate.

### GSH assay

GSH level analysis was performed using the GSH kit (Solarbio) following the manufacturer’s protocol. The experiments were executed in triplicate.

### MDA assay

MDA level measurement was performed using the MDA kit (Biosharp) following the manufacturer’s protocol. The experiments were executed in triplicate.

### Cell proliferation analysis

CCK-8 and clone formation assays were used to assess cell proliferation. CCK-8 analysis was performed using CCK-8 Kit (Abbkine) following the manufacturer’s protocol. In addition, SW480 and HCT116 cells were seeded in 6-well plates and transfected with siUSP52 or siControl. After 10 days, the samples were washed twice with PBS, fixed with 4% paraformaldehyde for 20 min, and stained with crystal violet for 20 min. The experiments were executed in triplicate.

### Cell migration and invasion analysis

Wound healing and transwell assays were performed to detect cell migration and invasion. SW480 and HCT116 cells were seeded in 6-well plates and transfected with siUSP52 or siControl. When the cell density reached 80%, we made a vertical scratch across the cell monolayer using a 10 μl pipette tip, washed with PBS, and added FBS-free medium. The wound images at 0 h and 24 h were captured using an inverted fluorescence microscope (Olympus). In transwell assays, after 24 h, cells were washed and counted. They were placed into a 24-well plate at a density of 1 × 10^5^ cells per insert. The medium containing 20% FBS was add in each well, while adding FBS-free medium in the inserts. After 48 h, the insert was washed twice with PBS, fixed with 4% paraformaldehyde for 20 min, and stained with crystal violet for 20 min. The photographs were taken using the aforementioned microscope. The experiments were executed in triplicate.

### Clinical samples

A total of 30 CRC tissues and adjacent normal tissues were obtained from Zhongnan Hospital of Wuhan University. All patients had received no anticancer treatment prior to biopsy collection. The human studies reported on in our research abide by the Declaration of Helsinki principles.

### Immunohistochemistry

Tissues were incubated with 4% paraformaldehyde. Subsequently, we carried out dehydration, embedding, and sectioning. Antigen retrieval was performed by heating the sections in 0.01 M citrate buffer (pH 6.0) at 105 °C for 10 min. Subsequently, the slides were blocked with 1% FBS and then incubated overnight at 4 °C with the primary antibody and the secondary antibody conjugated with horseradish peroxidase. After incubation, the immunocomplex was revealed with 3,3′-diaminobenzidine, and the nuclei were counterstained with hematoxylin. We then performed dehydration and mounted slides with neutral balsam. Images were scanned and captured using an inverted fluorescence microscope (Olympus). The primary antibodies and secondary antibodies used in the present study are shown in Table S6. The IHC score was analyzed using ImageJ (National Institutes of Health). The score criteria were as follows: The staining intensity of cells is graded into four levels (intensity: no staining: 0; weak staining: 1; intermediate staining: 2; and strong staining: 3). The percentage of positive cells is also graded into four levels (percentage: ≤25%: 1; 26%–50%: 2; 51%–75%: 3; and >75%: 4). The IHC score is equal to the staining intensity score multiplied by the percentage score of positive cells.

### Dual-luciferase reporter assay

SW480 and HCT116 cells were seeded in 24-well plates. TEAD luciferase reporter plasmid along with Renilla expression plasmid and the indicated plasmid were cotransfected into CRC cells. After 24 h transfection, luciferase activity was determined by a dual luciferase assay kit (Promega) according to the manufacturer’s protocol. The experiments were executed in triplicate.

### Tumor formation assays

HCT116 cells were stably transfected with shUSP52, shUSP52 + YAP, or empty vector. BALB/c nude mice (Moubaili) (4 weeks, male) were injected with approximately 7.5 × 10^6^ cells. Tumor formation in nude mice was supervised over 40 days. The tumor volume was computed by the formula: tumor volume = 0.5 × length × width^2^.

### Coimmunoprecipitation assay

SW480 and HCT116 cells were collected and lysed in 400 μl of NP-40 buffer (Beyotime) with complete protease inhibitor (MCE) on ice for 30 min. The cell lysate was centrifugation at 13,300 rpm for 25 min at 4 °C. The lysate supernatant (200 μl) was incubated overnight with ChIP-Grade Protein G Magnetic Beads (9006S; CST) and anti-YAP1 or anti-USP52 antibody at 4 °C on a rotating device. The corresponding immunoglobulin G (IgG) (30000-0-AP; Proteintech) was used as the negative control. Next, the magnetic beads were washed three times with immunoprecipitation lysate solution (Servicebio) and boiled for 10 min in 45 μl of 1× loading buffer. After centrifugation, the supernatant was collected and subjected to WB analysis.

### Protein stability assay

SW480 and HCT116 cells were seeded into 12-well plates and transfected with USP52 siRNA or siControl. After 48 h, the cells were processed with 100 μM cycloheximide (C7698; Sigma) for the indicated times. Samples were used for WB to evaluate YAP degradation.

### Polyubiquitination detection assay

To directly detect YAP polyubiquitination in cell extracts, HEK293 cells were transfected with the Ub, K6Ubi, K11Ubi, K27Ubi, K29Ubi, K33Ubi, K48Ubi, and K63Ubi plasmids together with the FLAG-USP52 plasmid and Myc-YAP or vector. After 48 h, the cells were processed with 10 μM MG132 (MCE; HY-13259) for 7 h and lysed by the denatured buffer (6 M guanidine–HCl, 0.1 M Na_2_HPO_4_/NaH_2_PO_4_, and 10 mM imidazole) (74). Total protein was then collected, and lysates were precleared with IgG and 20 μl of protein A/G (Biolinkedin) for 2 h. The supernatant was harvested and immunoprecipitated with an anti-Myc antibody. WB with an anti-HA antibody was performed to detect YAP polyubiquitination.

### Nuclear-cytoplasmic fractionation assay

SW480 and HCT116 cells were seeded into 10 cm^2^ dish and transfected with siUSP52 or siControl. After 72 h, this assay was performed using nuclear and cytoplasmic proteins extraction kit (Beyotime) following the manufacturer’s protocol.

### Immunotherapy analysis

MC38 cells were stably transfected with shUSP52 or empty vector for this study. For the *in vivo* immunotherapy experiment, C57BL/6 mice (Moubaili, Wuhan, China) (5 weeks, male) were injected with approximately 5 × 10^5^ cells. The administration of treatments began on the 10th day. At this point, the average tumor volume reached approximately 100 mm^3^. It is worth noting that the mice carrying the tumors were subjected to injections of IgG monoclonal antibody (mAb) (200 μg/mouse) or anti-PD-L1 mAb (200 μg/mouse), with a dosage regimen of once every 3 days (75). The IgG mAb and anti-PD-L1 mAb diluted with PBS before used. The IgG mAb used in this study was obtained from BioXcell (BE0093). The anti-PD-L1 mAb used in this study was obtained from BioXcell (BE0101). The experimental endpoint was set at the 24th day. At the conclusion of the experiment, tumor volume and weight were measured, and tumor tissues were collected for further analysis and experimentation.

### Statistical analysis

Statistical analyses were conducted with GraphPad Prism 10 (GraphPad). Differences between groups were analyzed by *t* test for comparisons between two groups or one-way ANOVA for multiple comparisons. The χ^2^ test was applied for categorical variables. The correlation of measurements was determined using Pearson’s correlation analysis. Measurement data are presented as the mean ± SD, and *p* < 0.05 was considered to indicate a significant difference.

## Data availability

All public datasets enrolled in this study could download from the TCGA database (https://www.cancer.gov/ccg/research/genome-sequencing/tcga). All data generated or analyzed during this study are included in the supporting information files of this article. Participants gave informed consent for data sharing.

## Ethics approval and consent to participate

The study was reviewed and approved by the Medical Ethics Committee of the Zhongnan Hospital of Wuhan University Institutional Review Board (Approval no.: 2025001K). The human studies reported in our research abide by the Declaration of Helsinki principles. This study has obtained informed consent from all patients. All the methods were carried out in accordance with the relevant guidelines under the ethical approval and consent to participate section.

## Supporting information

This article contains supporting information.

## Conflict of interest

The authors declare that they have no conflicts of interest with the contents of this article.

## References

[1] Sung H., Ferlay J., Siegel R.L., Laversanne M., Soerjomataram I., Jemal A. (2021). Global cancer statistics 2020: GLOBOCAN estimates of incidence and mortality worldwide for 36 cancers in 185 countries. CA Cancer J. Clin..

[2] Morgan E., Arnold M., Gini A., Lorenzoni V., Cabasag C.J., Laversanne M. (2023). Global burden of colorectal cancer in 2020 and 2040: incidence and mortality estimates from GLOBOCAN. Gut.

[3] Howren A., Sayre E.C., Loree J.M., Gill S., Brown C.J., Raval M.J. (2021). Trends in the incidence of young-onset colorectal cancer with a focus on years approaching screening age: a population-based longitudinal study. J. Natl. Cancer Inst..

[4] Dickinson B.T., Kisiel J., Ahlquist D.A., Grady W.M. (2015). Molecular markers for colorectal cancer screening. Gut.

[5] Sun T., Liu Z., Yang Q. (2020). The role of ubiquitination and deubiquitination in cancer metabolism. Mol. Cancer.

[6] Dewson G., Eichhorn P.J.A., Komander D. (2023). Deubiquitinases in cancer. Nat. Rev. Cancer.

[7] Antao A.M., Tyagi A., Kim K.S., Ramakrishna S. (2020). Advances in deubiquitinating enzyme inhibition and applications in cancer therapeutics. Cancers (Basel).

[8] Mennerich D., Kubaichuk K., Kietzmann T. (2019). DUBs, hypoxia, and cancer. Trends Cancer.

[9] Bett J.S., Ibrahim A.F., Garg A.K., Kelly V., Pedrioli P., Rocha S. (2013). The P-body component USP52/PAN2 is a novel regulator of HIF1A mRNA stability. Biochem. J..

[10] Yang S., Liu L., Cao C., Song N., Wang Y., Ma S. (2018). USP52 acts as a deubiquitinase and promotes histone chaperone ASF1A stabilization. Nat. Commun..

[11] Gao M., Guo G., Huang J., Kloeber J.A., Zhao F., Deng M. (2020). USP52 regulates DNA end resection and chemosensitivity through removing inhibitory ubiquitination from CtIP. Nat. Commun..

[12] Liu J., Luo Y., Chen S., Wang G., Jin W., Jiang W. (2024). Deubiquitylase USP52 promotes bladder cancer progression by modulating ferroptosis through stabilizing SLC7A11/xCT. Adv. Sci. (Weinh).

[13] Zhou J., Nie H., Yang X., Wang F., Yu P., Yu Y. (2024). Ubiquitin-specific protease 52 as a prognostic biomarker correlates with tumor microenvironment and therapy response in colorectal cancer. Oncology.

[14] Khan S.U., Fatima K., Aisha S., Malik F. (2024). Unveiling the mechanisms and challenges of cancer drug resistance. Cell Commun Signal.

[15] Liu S., Zhang X., Wang W., Li X., Sun X., Zhao Y. (2024). Metabolic reprogramming and therapeutic resistance in primary and metastatic breast cancer. Mol. Cancer.

[16] Yang S., Hu C., Chen X., Tang Y., Li J., Yang H. (2024). Crosstalk between metabolism and cell death in tumorigenesis. Mol. Cancer.

[17] Dixon S.J., Lemberg K.M., Lamprecht M.R., Skouta R., Zaitsev E.M., Gleason C.E. (2012). Ferroptosis: an iron-dependent form of nonapoptotic cell death. Cell.

[18] Chen Z., Wang W., Abdul Razak S.R., Han T., Ahmad N.H., Li X. (2023). Ferroptosis as a potential target for cancer therapy. Cell Death Dis.

[19] Lei G., Zhuang L., Gan B. (2022). Targeting ferroptosis as a vulnerability in cancer. Nat. Rev. Cancer.

[20] Tong X., Tang R., Xiao M., Xu J., Wang W., Zhang B. (2022). Targeting cell death pathways for cancer therapy: recent developments in necroptosis, pyroptosis, ferroptosis, and cuproptosis research. J. Hematol. Oncol..

[21] Zhang C., Liu X., Jin S., Chen Y., Guo R. (2022). Ferroptosis in cancer therapy: a novel approach to reversing drug resistance. Mol. Cancer.

[22] Lin Z., Liu J., Long F., Kang R., Kroemer G., Tang D. (2022). The lipid flippase SLC47A1 blocks metabolic vulnerability to ferroptosis. Nat. Commun..

[23] Driskill J.H., Pan D. (2023). Control of stem cell renewal and fate by YAP and TAZ. Nat. Rev. Mol. Cell Biol.

[24] Sladitschek-Martens H.L., Guarnieri A., Brumana G., Zanconato F., Battilana G., Xiccato R.L. (2022). YAP/TAZ activity in stromal cells prevents ageing by controlling cGAS-STING. Nature.

[25] Dey A., Varelas X., Guan K.L. (2020). Targeting the Hippo pathway in cancer, fibrosis, wound healing and regenerative medicine. Nat. Rev. Drug Discov..

[26] Rausch V., Hansen C.G. (2020). The Hippo pathway, YAP/TAZ, and the plasma membrane. Trends Cell Biol.

[27] Magesh S., Cai D. (2022). Roles of YAP/TAZ in ferroptosis. Trends Cell Biol.

[28] Gu Y., Wu S., Fan J., Meng Z., Gao G., Liu T. (2024). CYLD regulates cell ferroptosis through Hippo/YAP signaling in prostate cancer progression. Cell Death Dis.

[29] Fang K., Du S., Shen D., Xiong Z., Jiang K., Liang D. (2022). SUFU suppresses ferroptosis sensitivity in breast cancer cells via Hippo/YAP pathway. iScience.

[30] Goodman R.S., Johnson D.B., Balko J.M. (2023). Corticosteroids and cancer immunotherapy. Clin. Cancer Res..

[31] Cella D., Motzer R.J., Suarez C., Blum S.I., Ejzykowicz F., Hamilton M. (2022). Patient-reported outcomes with first-line nivolumab plus cabozantinib versus sunitinib in patients with advanced renal cell carcinoma treated in CheckMate 9ER: an open-label, erceptin, phase 3 trial. Lancet Oncol..

[32] Doki Y., Ajani J.A., Kato K., Xu J., Wyrwicz L., Motoyama S. (2022). Nivolumab combination therapy in advanced esophageal squamous-cell carcinoma. N. Engl. J. Med..

[33] Socinski M.A., Jotte R.M., Cappuzzo F., Orlandi F., Stroyakovskiy D., Nogami N. (2018). Impower150 study group. Atezolizumab for first-line treatment of metastatic Nonsquamous NSCLC. N. Engl. J. Med..

[34] Robert C., Ribas A., Schachter J., Arance A., Grob J.J., Mortier L. (2019). Pembrolizumab versus ipilimumab in advanced melanoma (KEYNOTE-006): post-hoc 5-year results from an open-label, multicentre, randomised, controlled, phase 3 study. Lancet Oncol..

[35] Najafi S., Majidpoor J., Mortezaee K. (2022). The impact of microbiota on PD-1/PD-L1 inhibitor therapy outcomes: a focus on solid tumors. Life Sci..

[36] Park J.J., Thi E.P., Carpio V.H., Bi Y., Cole A.G., Dorsey B.D. (2021). Checkpoint inhibition through small molecule-induced internalization of programmed death-ligand 1. Nat. Commun..

[37] Xu C., Sun S., Johnson T., Qi R., Zhang S., Zhang J. (2021). The glutathione peroxidase Gpx4 prevents lipid peroxidation and ferroptosis to sustain Treg cell activation and suppression of antitumor immunity. Cell Rep.

[38] Guo P., Wang L., Shang W., Chen J., Chen Z., Xiong F. (2020). Intravesical *in situ* immunostimulatory gel for triple therapy of bladder cancer. ACS Appl. Mater. Inter..

[39] Chen J.J., Galluzzi L. (2018). Fighting resilient cancers with iron. Trends Cell Biol..

[40] Roh J.L., Kim E.H., Jang H., Shin D. (2017). Nrf2 inhibition reverses the resistance of cisplatin-resistant head and neck cancer cells to artesunate-induced ferroptosis. Redox Biol..

[41] Tang R., Xu J., Zhang B., Liu J., Liang C., Hua J. (2020). Ferroptosis, necroptosis, and pyroptosis in anticancer immunity. J. Hematol. Oncol..

[42] Pan B., Yang Y., Li J., Wang Y., Fang C., Yu F.X. (2020). USP47-mediated deubiquitination and stabilization of YAP contributes to the progression of colorectal cancer. Protein Cell.

[43] Tian Z., Xu C., He W., Lin Z., Zhang W., Tao K. (2023). The deubiquitinating enzyme USP19 facilitates hepatocellular carcinoma progression through stabilizing YAP. Cancer Lett..

[44] Cappadocia L., Lima C.D. (2018). Ubiquitin-like protein conjugation: structures, chemistry, and mechanism. Chem. Rev..

[45] Jiang X., Stockwell B.R., Conrad M. (2021). Ferroptosis: mechanisms, biology and role in disease. Nat. Rev. Mol. Cell Biol.

[46] Tang H., Li C., Zhang Y., Zheng H., Cheng Y., Zhu J. (2020). Targeted Manganese doped silica nano GSH-cleaner for treatment of Liver Cancer by destroying the intracellular redox homeostasis. Theranostics.

[47] Chen P., Li X., Zhang R., Liu S., Xiang Y., Zhang M. (2020). Combinative treatment of β-elemene and cetuximab is sensitive to KRAS mutant colorectal cancer cells by inducing ferroptosis and inhibiting epithelial-mesenchymal transformation. Theranostics.

[48] Guan X., Wang Y., Yu W., Wei Y., Lu Y., Dai E. (2024). Blocking ubiquitin-specific protease 7 induces ferroptosis in gastric cancer via targeting stearoyl-CoA desaturase. Adv. Sci. (Weinh).

[49] Li H., Sun Y., Yao Y., Ke S., Zhang N., Xiong W. (2024). USP8-governed GPX4 homeostasis orchestrates ferroptosis and cancer immunotherapy. Proc. Natl. Acad. Sci. U S A..

[50] Torres M.P., Lee M.J., Ding F., Purbeck C., Kuhlman B., Dokholyan N.V. (2009). G protein mono-ubiquitination by the Rsp5 ubiquitin ligase. J. Biol. Chem..

[51] Kwon Y.T., Ciechanover A. (2017). The ubiquitin code in the ubiquitin-proteasome system and autophagy. Trends Biochem. Sci..

[52] Rahman S., Wolberger C. (2024). Breaking the K48-chain: linking ubiquitin beyond protein degradation. Nat. Struct. Mol. Biol..

[53] Zhang W., Luo J., Xiao Z., Zang Y., Li X., Zhou Y. (2022). USP36 facilitates esophageal squamous carcinoma progression via stabilizing YAP. Cell Death Dis.

[54] Mo H., Li R., Yang N., Han J., Xiao X., Zhang Y. (2024). USP40 promotes hepatocellular carcinoma progression through a YAP/USP40 positive feedback loop. Cancer Lett..

[55] Chen Z.J., Sun L.J. (2009). Nonproteolytic functions of ubiquitin in cell signaling. Mol. Cell.

[56] Yao F., Zhou Z., Kim J., Hang Q., Xiao Z., Ton B.N. (2018). SKP2- and OTUD1-regulated non-proteolytic ubiquitination of YAP promotes YAP nuclear localization and activity. Nat. Commun..

[57] Panda D.K., Bai X., Zhang Y., Stylianesis N.A., Koromilas A.E., Lipman M.L. (2022). SCF-SKP2 E3 ubiquitin ligase links mTORC1/ER stress/ISR with YAP activation in murine renal cystogenesis. J. Clin. Invest..

[58] Liu M., Yan M., Lv H., Wang B., Lv X., Zhang H. (2020). Macrophage K63-linked ubiquitination of YAP promotes its nuclear localization and exacerbates atherosclerosis. Cell Rep.

[59] Matsumoto M.L., Wickliffe K.E., Dong K.C., Yu C., Bosanac I., Bustos D. (2010). K11-linked polyubiquitination in cell cycle control revealed by a K11 linkage-specific antibody. Mol. Cell.

[60] Tracz M., Bialek W. (2021). Beyond K48 and K63: non-canonical protein ubiquitination. Cell Mol Biol Lett.

[61] Liu D., Li Q., Zang Y., Li X., Li Z., Zhang P. (2023). USP1 modulates hepatocellular carcinoma progression via the Hippo/TAZ axis. Cell Death Dis.

[62] Wang Z., Shen N., Wang Z., Yu L., Yang S., Wang Y. (2024). TRIM3 facilitates ferroptosis in non-small cell lung cancer through promoting SLC7A11/xCT K11-linked ubiquitination and degradation. Cell Death Differ.

[63] Zhong B., Du J., Liu F., Sun S. (2025). The role of yes-associated protein in inflammatory diseases and cancer. MedComm.

[64] Zhou X., Li Y., Wang W., Wang S., Hou J., Zhang A. (2020). Regulation of Hippo/YAP signaling and esophageal squamous carcinoma progression by an E3 ubiquitin ligase PARK2. Theranostics.

[65] Chen Y.A., Lu C.Y., Cheng T.Y., Pan S.H., Chen H.F., Chang N.S. (2019). WW domain-containing proteins YAP and TAZ in the Hippo pathway as key regulators in stemness maintenance, tissue homeostasis, and tumorigenesis. Front. Oncol..

[66] Yang W.H., Chi J.T. (2019). Hippo pathway effectors YAP/TAZ as novel determinants of ferroptosis. Mol. Cell Oncol..

[67] Gao R., Kalathur R.K.R., Coto-Llerena M., Ercan C., Buechel D., Shuang S. (2021). YAP/TAZ and ATF4 drive resistance to Sorafenib in hepatocellular carcinoma by preventing ferroptosis. EMBO Mol. Med..

[68] Zhang F., Huang B., Xu Y., Cao G., Shen M., Liu C. (2025). MISP suppresses ferroptosis via MST1/2 kinases to facilitate YAP activation in non-small cell lung cancer. Adv. Sci. (Weinh).

[69] Gong J., Chehrazi-Raffle A., Reddi S., Salgia R. (2018). Development of PD-1 and PD-L1 inhibitors as a form of cancer immunotherapy: a comprehensive review of registration trials and future considerations. J. Immunother. Cancer.

[70] Boisgerault N., Bertrand P. (2023). Inside PD-1/PD-L1,2 with their inhibitors. Eur. J. Med. Chem..

[71] Cheng B., Pan W., Xiao Y., Ding Z., Zhou Y., Fei X. (2024). HDAC-targeting epigenetic modulators for cancer immunotherapy. Eur. J. Med. Chem..

[72] Zhang Z., Zhang Y., Xia S., Kong Q., Li S., Liu X. (2020). Gasdermin E suppresses tumour growth by activating anti-tumour immunity. Nature.

[73] Liang J.Y., Wang D.S., Lin H.C., Chen X.X., Yang H., Zheng Y. (2020). A novel ferroptosis-related gene signature for overall survival prediction in patients with hepatocellular carcinoma. Int. J. Biol. Sci..

[74] Chan C.H., Li C.F., Yang W.L., Gao Y., Lee S.W., Feng Z. (2012). The Skp2-SCF E3 ligase regulates Akt ubiquitination, glycolysis, erceptin sensitivity, and tumorigenesis. Cell.

[75] Tang Y., Zhou C., Li Q., Cheng X., Huang T., Li F. (2022). Targeting depletion of myeloid-derived suppressor cells potentiates PD-L1 blockade efficacy in gastric and colon cancers. Oncoimmunology.

