# Supplementary material for: USP52 inhibits cell ferroptosis via Hippo–YAP pathway and blocks immunotherapy in colorectal cancer
Source: J Biol Chem. 2025 Sep 15;301(11):110725. doi: 10.1016/j.jbc.2025.110725 (PMC12554183; doi:10.1016/j.jbc.2025.110725)
Supplement: Supporting Figures [file mmc2.docx]

**Supplementary Figures**


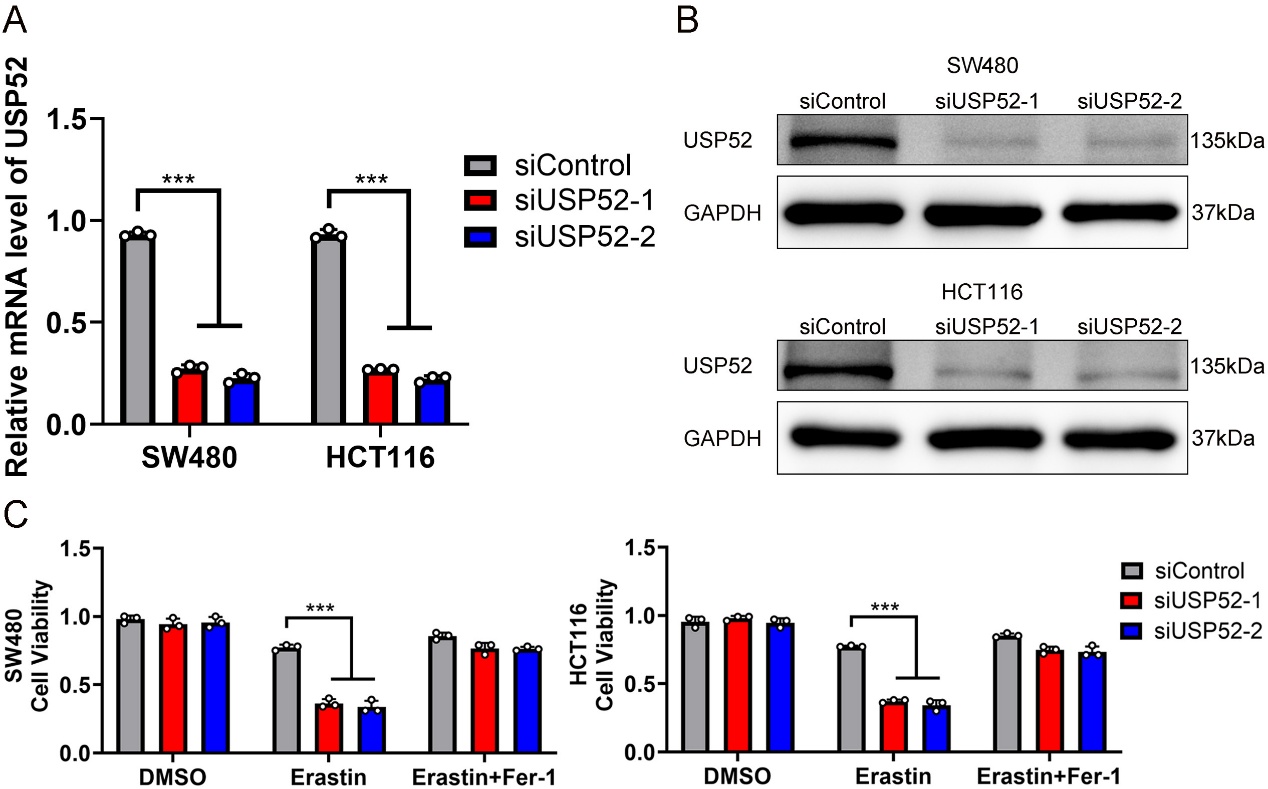


**Figure S1** Evaluation of USP52 knockdown efficiency and quantitative analysis of cell viability. **A:** The expression of USP52 was significantly decreased using siRNA targeting USP52 at the transcriptional level; **B:** The expression of USP52 was markedly decreased using siRNA targeting USP52 at the translational level; **C:** Quantitative analysis of cell viability revealed that USP52-silenced SW480 and HCT116 cells increased the sensitivity to ferroptosis inducer Erastin, and this effect could be reversed by ferroptosis inhibitor Fer-1. Small interfering RNAs, siRNA. ***P value < 0.001.


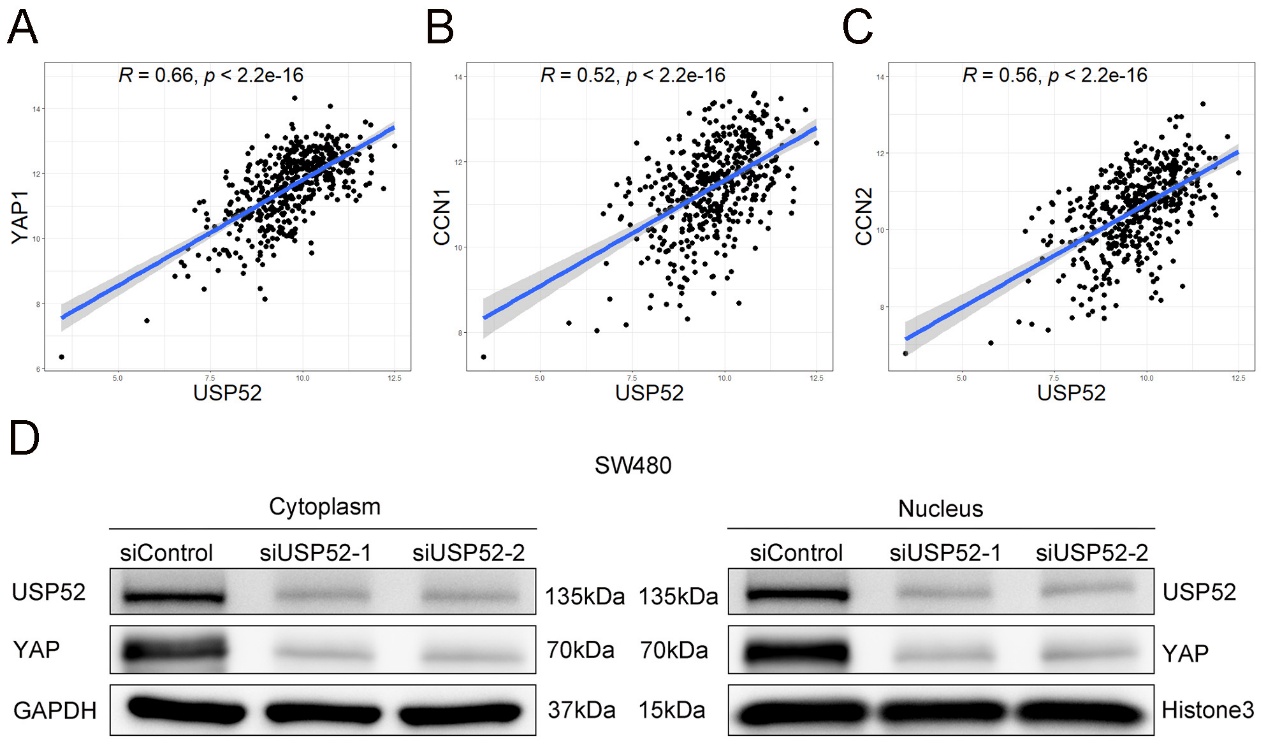


**Figure S2** Correlation analysis and nuclear-cytoplasmic fractionation assay. **A:** Correlation analysis revealed a positive correlation between USP52 and YAP1; **B:** A positive correlation between USP52 and CCN1; **C:** A positive correlation between USP52 and CCN2; **D:** The nuclear-cytoplasmic fractionation assay demonstrated that USP52 depletion led to a decrease in the expression of YAP in the cytoplasm and the nucleus.


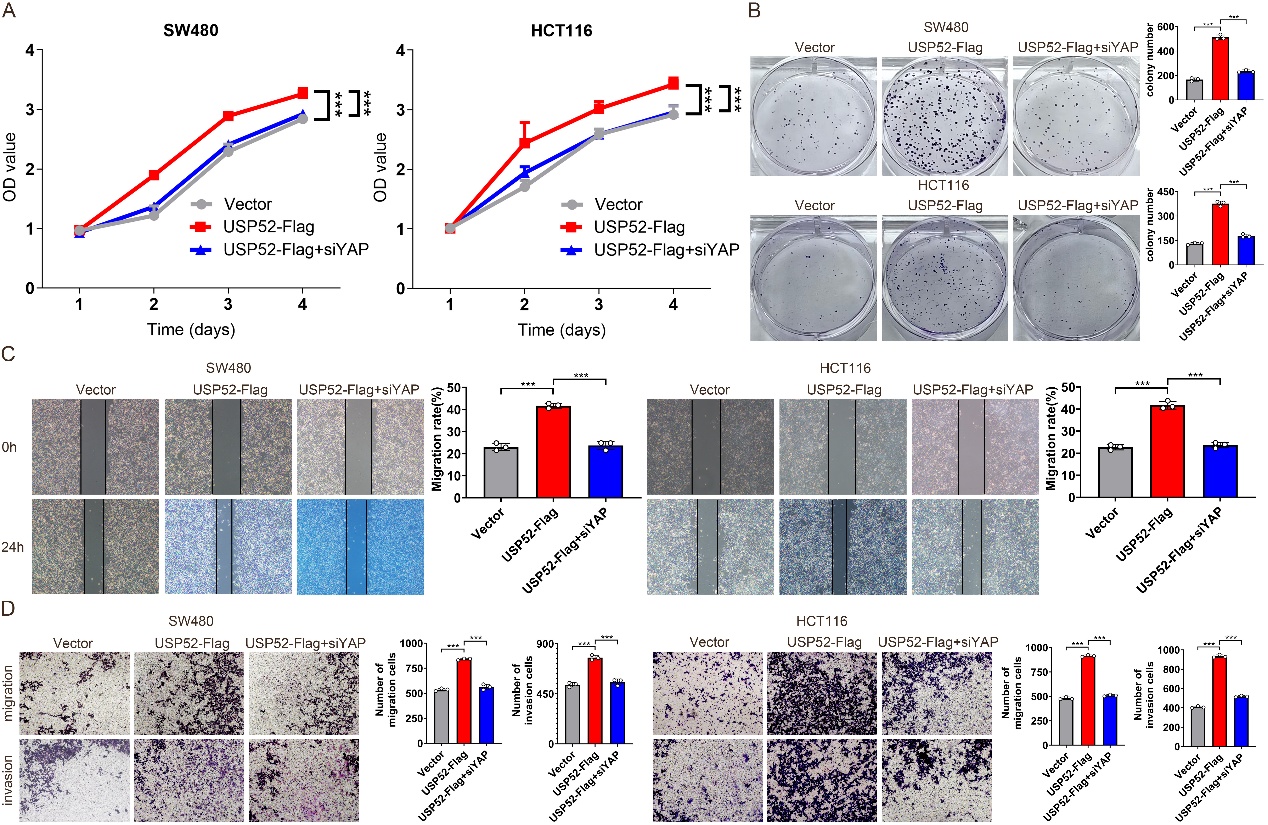


**Figure S3** USP52 promoted the progression of CRC through the Hippo/YAP pathway. **A:** The CCK8 assay demonstrated that USP52 overexpression significantly promoted the proliferation of SW480 and HCT116 cells, and this effect could be reversed by YAP knockdown; **B:** The colony formation assay revealed that USP52 overexpression noticeably increased the number of cell colonies, which could be attenuated by YAP depletion; **C:** The wound healing assay indicated that USP52 overexpression significantly promoted cell migration, which could be partially reversed by YAP inhibition; **D:** The transwell assay found that USP52 overexpression markedly facilitated cell migration and invasion, which was partially attenuated by YAP knockdown. Colorectal cancer, CRC; Cell Counting Kit-8, CCK8. ***P value < 0.001.


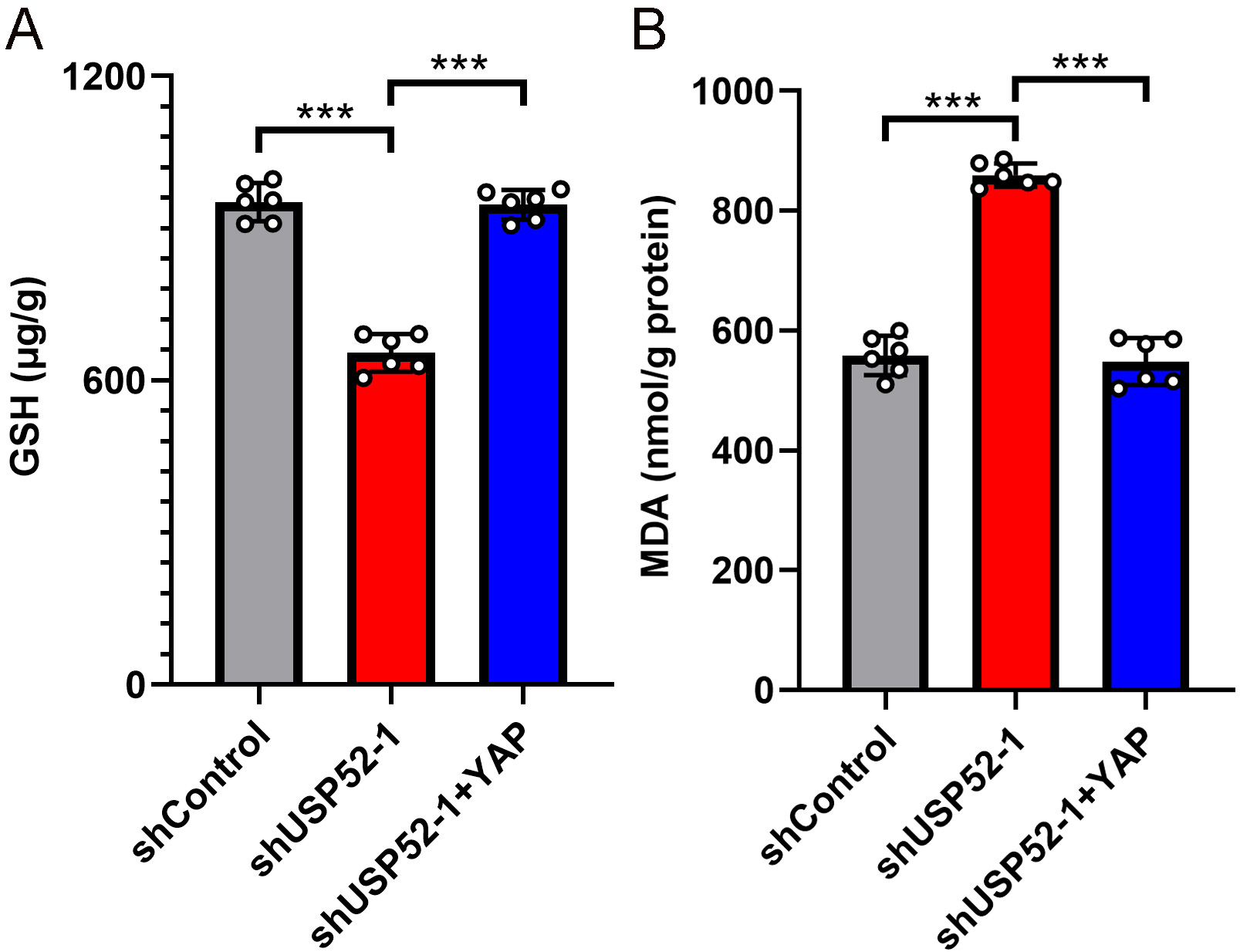


**Figure S4** USP52 inhibited ferroptosis through Hippo/YAP axis in vivo. **A:** GSH assays revealed that USP52 knockdown significantly decreased GSH levels in tumor tissues, while YAP overexpression partially attenuated this inhibition effect; **B:** MDA assays indicated that USP52 depletion markedly increased MDA levels in tumor tissues, which was reversed by YAP overexpression. Glutathione, GSH; malondialdehyde, MDA. ***P value < 0.001.
